# High ALDH Activity Identifies Chemotherapy-Resistant Ewing's Sarcoma Stem Cells That Retain Sensitivity to EWS-FLI1 Inhibition

**DOI:** 10.1371/journal.pone.0013943

**Published:** 2010-11-11

**Authors:** Ola Awad, Jason T. Yustein, Preeti Shah, Naheed Gul, Varalakshmi Katuri, Alison O'Neill, Yali Kong, Milton L. Brown, Jeffrey A. Toretsky, David M. Loeb

**Affiliations:** 1 Division of Pediatric Oncology, Department of Oncology, Sidney Kimmel Comprehensive Cancer Center, Johns Hopkins University, Baltimore, Maryland, United States of America; 2 Department of Oncology, Lombardi Comprehensive Cancer Center, Georgetown University, Washington, D.C., United States of America; 3 Department of Pediatrics, Lombardi Comprehensive Cancer Center, Georgetown University, Washington, D.C., United States of America; University of Hong Kong, China

## Abstract

**Background:**

Cancer stem cells are a chemotherapy-resistant population capable of self-renewal and of regenerating the bulk tumor, thereby causing relapse and patient death. Ewing's sarcoma, the second most common form of bone tumor in adolescents and young adults, follows a clinical pattern consistent with the Cancer Stem Cell model – remission is easily achieved, even for patients with metastatic disease, but relapse remains frequent and is usually fatal.

**Methodology/Principal Findings:**

We have isolated a subpopulation of Ewing's sarcoma cells, from both human cell lines and human xenografts grown in immune deficient mice, which express high aldehyde dehydrogenase (ALDH^high^) activity and are enriched for clonogenicity, sphere-formation, and tumor initiation. The ALDH^high^ cells are resistant to chemotherapy *in vitro*, but this can be overcome by the ATP binding cassette transport protein inhibitor, verapamil. Importantly, these cells are not resistant to YK-4-279, a small molecule inhibitor of EWS-FLI1 that is selectively toxic to Ewing's sarcoma cells both *in vitro* and *in vivo*.

**Conclusions/Significance:**

Ewing's sarcoma contains an ALDH^high^ stem-like population of chemotherapy-resistant cells that retain sensitivity to EWS-FLI1 inhibition. Inhibiting the EWS-FLI1 oncoprotein may prove to be an effective means of improving patient outcomes by targeting Ewing's sarcoma stem cells that survive standard chemotherapy.

## Introduction

Ewing sarcoma family tumors (ESFT) are the second most common bone tumor in children and young adults. ESFT initially responds quite well to cytotoxic chemotherapy, but 30% of patients presenting with localized tumors develop recurrent disease and 65–80% of patients with metastatic ESFT die within 5 years of diagnosis [1], [2], [3]. The cancer stem cell (CSC) hypothesis provides a framework for explaining the discrepancy between response of ESFT to therapy and the poor survival rate. The origin of CSCs remains controversial, but whether they are derived directly from normal tissue stem cells, or from differentiated cells that have acquired stem cell properties through genetic mutations, these cells, like normal stem cells, can undergo asymmetric division and are capable of self renewal as well as giving rise to a population of differentiated tumor cells. CSCs have been identified in many hematologic and solid tumors, including both acute and chronic leukemias, brain tumors, breast cancer, and colon cancer [4], [5], [6], [7]. CSCs are postulated to be resistant to standard cytotoxic chemotherapeutic agents both in vitro and in vivo, and this resistance is postulated to be the major cause of treatment failure, as the surviving reservoir of stem cells repopulates the tumor leading to relapse. Nevertheless, not every tumor type adheres to the stem cell model [8]. Thus, there is an urgent need to identify and fully characterize ESFT stem cells, and to develop therapeutic approaches to target these cells and improve the survival of patients with recurrent or metastatic disease.

In 95% of cases, ESFT is associated with a translocation between the central exons of the EWS gene located on chromosome 22 and the central exons of an *ets* family gene; either FLI1 located on chromosome 11, or ERG located on chromosome 21 [9]. The resulting fusion protein acts as an aberrant transcription factor regulating genes involved in transformation. The EWS-FLI1 fusion protein is an excellent candidate for targeted therapy as its expression is limited to tumor cells and is crucial for initiation and maintenance of the tumor. Reducing EWS-FLI1 expression using antisense oligonucleotides or siRNA in cell lines results in decreased tumorigenicity both *in vitro* and *in vivo* [10], [11], [12]. Moreover, transduction of mesenchymal stem cells with EWS-FLI1 causes the development of tumors with an ESFT phenotype [13]. The mechanism by which EWS-FLI1 mediates neoplastic transformation is poorly understood. EWS-FLI1 has transcriptional regulatory activity, and a number of target genes have been identified that may play a role in neoplastic transformation [14], [15], [16], [17]. In addition to this activity, RNA helicase A (RHA) physically interacts with EWS-FLI1 and modulates oncogenesis, suggesting that this interaction is a promising therapeutic target [18]. We have developed a novel small molecule, YK-4-279, that inhibits the EWS-FLI1/RHA interaction, inducing apoptosis in ESFT cell lines and xenografts [19].

Aldehyde dehydrogenase (ALDH) has been proposed to be a marker of both normal and cancer stem cells [20] and has been used to identify CSC from colon, breast, and lung cancers, among others [21], [22], [23]. We have identified a CSC population in ESFT cell lines and xenografts based on high expression of ALDH. These cells fulfill the *in vitro* and *in vivo* criteria for stem cell activity including the ability to reconstitute a heterogeneous population, sphere- and colony-forming activity, and the ability to form tumors in immune deficient mice. We also found these cells to express high levels of stem cell-associated genes such as *bmi-1, nanog*, and *oct-4*. Moreover, these cells are relatively resistant to cytotoxic agents such as doxorubicin and etoposide. We then demonstrated that these cells express EWS-FLI1 and that, in contrast to cytotoxic drugs, they retain sensitivity to the small molecule inhibitor YK-4-279. Thus, our study utilizes a novel, molecularly targeted approach to target ESFT stem cells and bypass their relative chemoresistance, which may lead to new therapies to improve the survival of ESFT patients.

## Results

### ESFT cell lines and xenografts contain a small population of cells with high expression of aldehyde dehydrogenase

**Figure 1:**
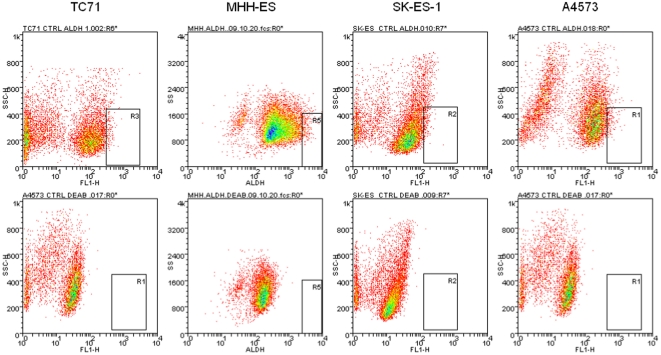
Aldehyde dehydrogenase (ALDH) expression in ESFT cell lines. The indicated cell lines were treated with Aldefluor reagent alone (top) or in the presence of the ALDH inhibitor DEAB (bottom), and then analyzed by FACS. Each cell line has an Aldefluor bright cell population that is undetectable in the presence of DEAB. In each panel, the box indicates a typical gate, containing approximately 2% of the cells, defined as ALDH^high^ and used for subsequent experiments. Of note, these cells also have low side scatter. These sorting experiments have been performed a minimum of three times on each cell line.

**Figure 2:**
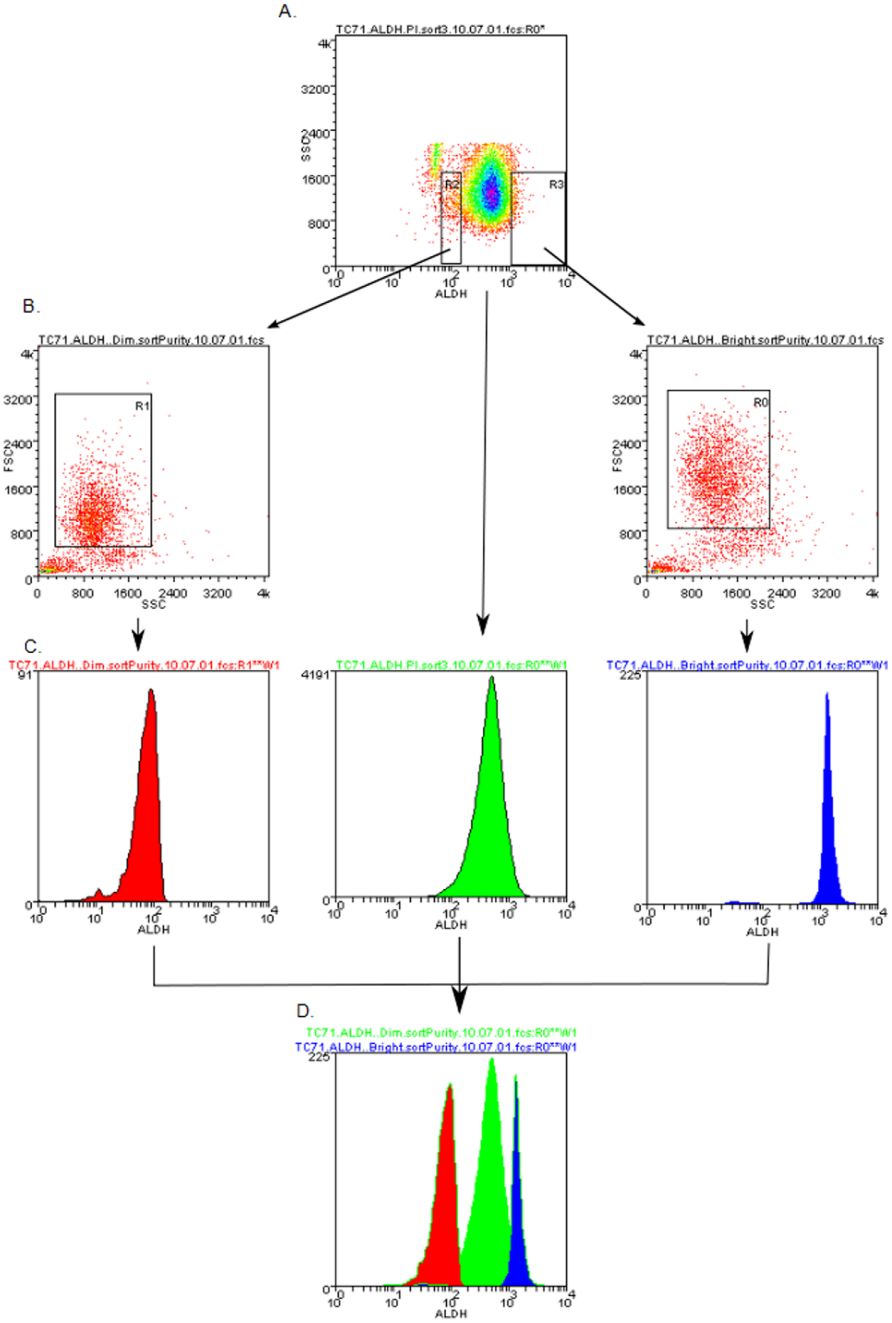
Evaluation of sorting efficiency. TC71 cells were incubated with the Aldefluor reagent and sorted as described (A). Viable (based on PI exclusion) ALDH^low^ and ALDH^high^ cells were isolated as indicated by the arrows (B). The viable cells from each population (as well as the entire population, in the center panel) were analyzed for Aldefluor-related fluorescence (C), and an overlay of the resulting histograms shows no appreciable overlap (D), demonstrating a sorting efficiency of >98%. This check was performed three times, and the data presented are from a representative experiment.

ALDH is one of a family of enzymes involved in several detoxifying pathways [24]. Elevated ALDH expression has recently been used to identify a rare stem cell-like population in several tumor types, including leukemia, brain, colon and breast cancer [25]. To determine whether ESFT cell lines contain a high ALDH expressing stem cell-like population, we used the Aldefluor reagent to test ALDH activity in five ESFT cell lines (TC71, MHH-ES, SK-ES-1, A4573, and RD-ES). Each of these cell lines contains a population of cells with fluorescence that is inhibited by the ALDH inhibitor diethylaminobenzaldehyde (DEAB; Figure 1). We observed a distribution of ALDH activity across the population of ESFT cells. We isolated the cells with the highest and lowest 2% of ALDH activity (designated ALDH^high^ and ALDH^low^) to test our hypothesis that although all cells express some amount of ALDH (and therefore will have DEAB-inhibitable fluorescence), the cells with the highest ALDH activity (ALDH^high^) are enriched for a stem cell phenotype. To determine the purity of our sorted cells, we isolated both ALDH^high^ and ALDH^low^ cells (Figure 2). Viable cells, as determined by exclusion of propidium iodide, were then reanalyzed using the original Aldefluor channel. This analysis revealed a sorting efficiency of >98% (Figure 2).

**Figure 3:**
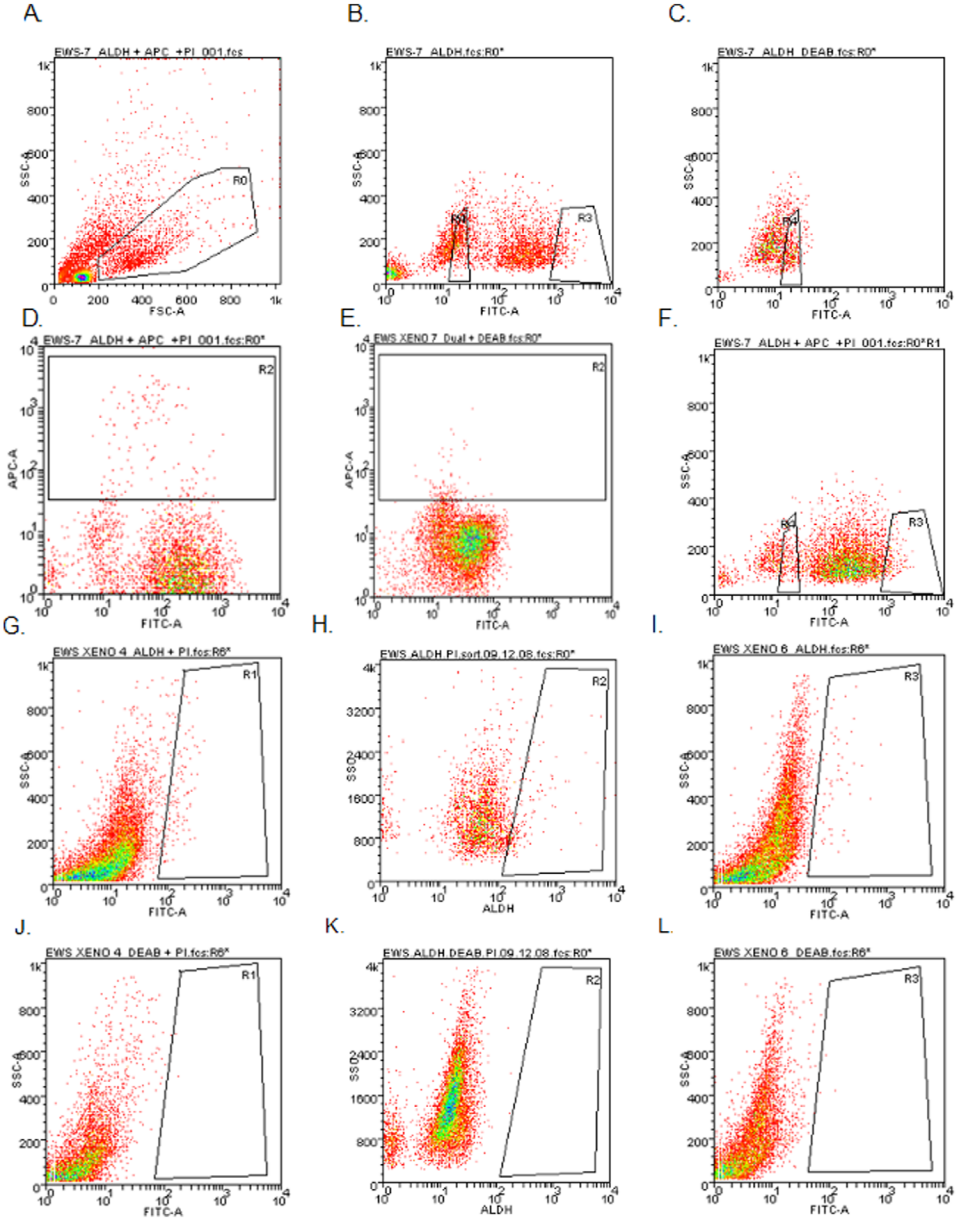
ALDH expression in primary human ESFT xenografts. Single cell suspensions were made from each of four early passage primary human ESFT xenografts. (A) The first step of purification was isolation of viable cells based on scatter and PI exclusion (enclosed in gate R0). Viable cells were stained with Aldefluor without (B) or with (C) DEAB. To exclude contamination with murine cells, single cell suspensions were incubated with an antibody against murine MHC after Aldefluor treatment without (D) or with (E) DEAB. FACS was used to eliminate the cells expressing mouse MHC (gate R2 in panels D and E), and cells were sorted for ALDH activity (F). In panel F, gate R3 is the ALDH^high^ cells and gate R4 is the ALDH^low^ cells. Three other xenografts were also analyzed by Aldefluor without (G, H, I) or with (J, K, L) DEAB.

In addition to cell lines, we also analyzed single cell suspensions prepared from three different early-passage ESFT xenografts of primary human tumors (generous gifts from Drs. C. Khanna and L. Helman, NIH), and we detected a similar distribution of ALDH expression in these cells (Figure 3). Initially, viable cells were isolated from the single cell suspension based forward scatter, side scatter, and exclusion of PI (Figure 3A, gate R0). Cell suspensions were stained with the Aldefluor reagent and sorted (Figure 3B). ALDH^low^ (Figure 3B, Gate R4) and ALDH^high^ (Figure 3B, Gate R3) cells were isolated for further analysis. As with the cell lines, DEAB served as a negative control (Figure 3C). To exclude contamination with murine cells, single cell suspensions were incubated with the Aldefluor reagent, and then with an antibody against the murine major histocompatibility complex (MHC) proteinH2Kd. FACS was used to eliminate the cells expressing mouse MHC (which accounted for 2.5% of the total; Figure 3D, gate R2; Figure 3E shows the effect of including DEAB). Subsequent sorting for ALDH activity (Figure 3F) gave results indistinguishable from experiments from which murine cells were not excluded. An ALDH^high^ population was also seen in three other early passage xenografts (Figures 3G, H, and I, with DEAB control in J, K, and L).

**Figure 4:**
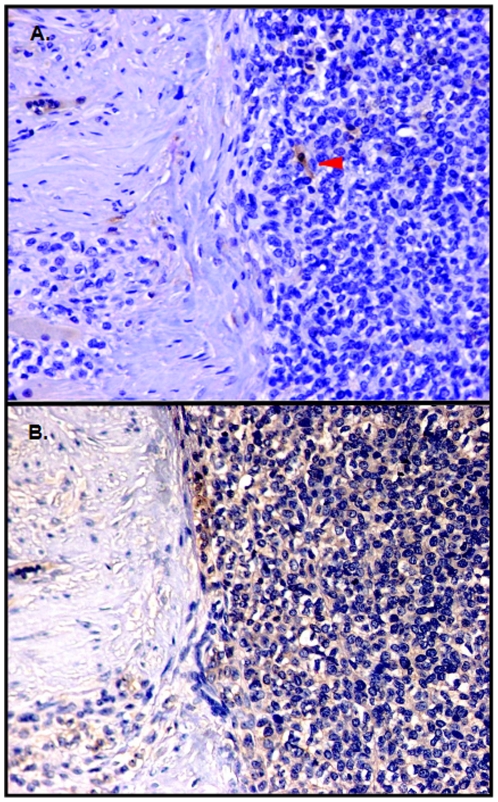
ALDH expression in primary ESFT biopsy samples. (A). Representative section of a primary Ewing's sarcoma at low power (20×) showing expression of ALDH1, as revealed by immunohistochemistry. The red triangle indicates the cell with the strongest staining. (B). An adjacent section from the same biopsy specimen demonstrating CD99 expression. In both images, positive staining is indicated by the brown color. Samples were counterstained with hematoxylin to show general tumor histology.

Because ESFTs are diagnosed by needle biopsy and not resected until after administration of neoadjuvant chemotherapy, adequate numbers of cells from newly diagnosed tumors could not be obtained to verify these findings in primary patient samples; however, we performed immunohistochemistry to evaluate ALDH1 expression in a panel of twenty-two biopsy specimens obtained from a total of 10 patients with ESFT (mean 2.2 biopsy samples per patient, with a range of 1–4). We found variable ALDH protein expression in each specimen, with a gradient of staining, including cells lacking expression, cells with modest amounts of staining, and a small minority with very high intensity staining (Figure 4A), consistent with our flow cytometry results from cell lines and xenografts. Expression was quantified using the FRIDA image analysis software package. An average of 0.7% (±1.3) of cells stained for ALDH, and 99.3% (±1.3) of cells were negative (p<0.0001). Staining adjacent sections with CD99, a marker for ESFT cells (Figure 4B), confirmed that the ALDH-positive cells were indeed tumor cells.

### ALDH^high^ cells are enriched for clonogenic and sphere forming activity

**Figure 5:**
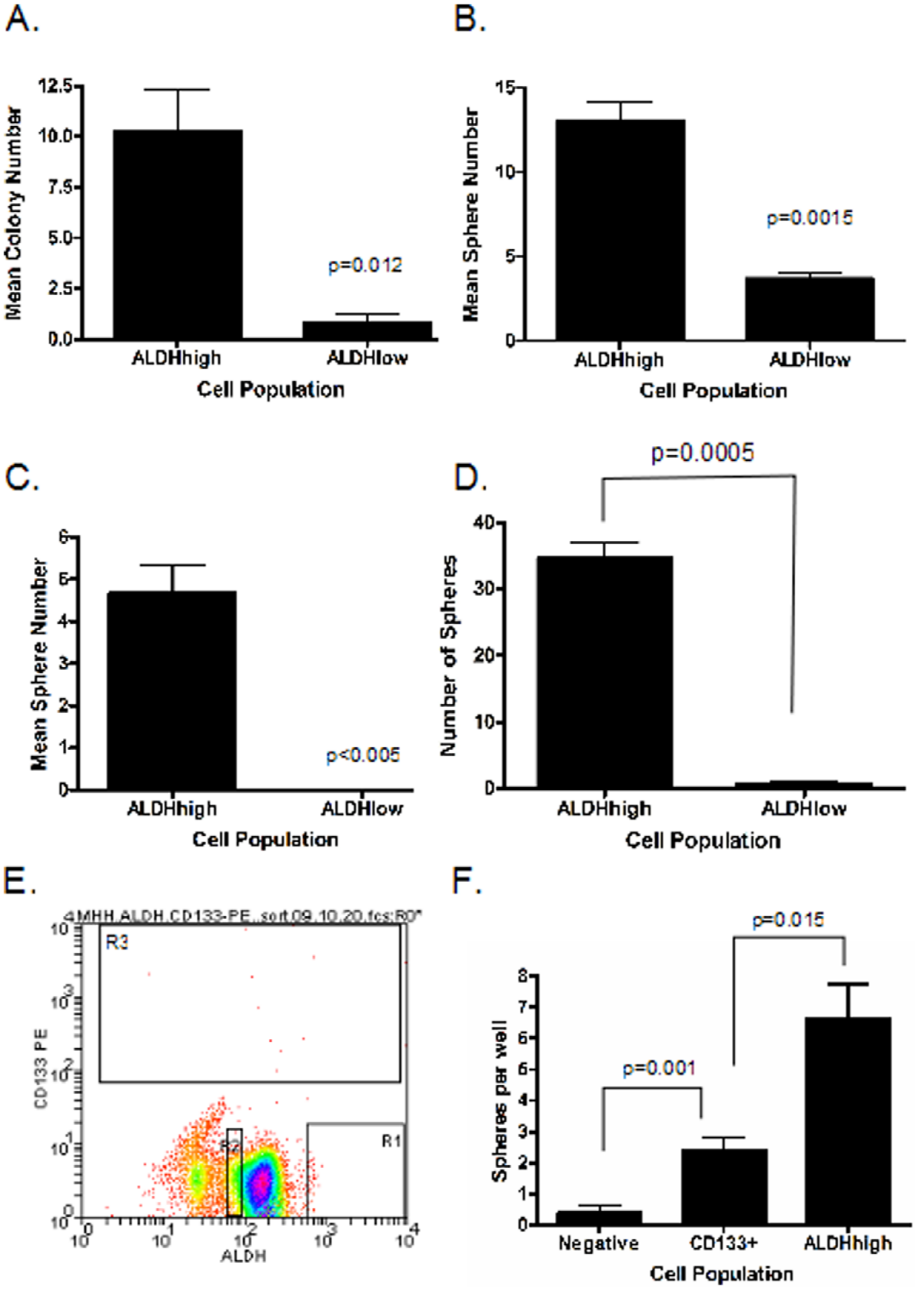
Clonogenic and sphere forming activity of ALDH ^high^ cells. A) ALDH^high^ and ALDH^low^ TC71 cells were plated in soft agar at 1000 cells/well. Data from 3 independent experiments represent average colony count/well after 14 days. B) ALDH^high^ and ALDH^low^ TC71 cells were plated in Mesencult at 1000 cells/well for 7 days. Data shown is the average sphere count from a representative experiment performed in triplicate wells. C) Average sphere count from ALDH^high^ and ALDH^low^ cells isolated from an ESFT xenograft. 3000 cell/well in triplicate wells were cultured in Mesencult for 14 days. Bars = SEM (D) ALDH^high^ and ALDH^low^ cells isolated from a second ESFT xenograft, this one with contaminating murine cells excluded as shown in Figure 3 were cultured in Mesencult for 14 days in triplicate wells with 3000 cells/well. Bars = SEM. (E) MHH-ES cells were incubated with the Aldefluor reagent followed by PE-labeled anti-CD133 and then were sorted for ALDH and CD133 expression simultaneously. Only a small number of CD133-positive cells are seen, and these are evenly distributed across the ALDH spectrum (gate R3). (F) CD133-positive cells, ALDH^high^ cells, and control cells (CD133-negative, ALDH^low^) were plated in Mesencult at 1000 cells/well for 7 days. Data shown is the average sphere count from a representative experiment performed in triplicate wells. Bars = SEM. Each experiment was performed a minimum of three times. In each panel, the p value shows the significance of the difference between the indicated populations, as determined by a one-tailed t test.

Clonogenic activity in soft agar has been proposed as an *in vitro* measure of tumor initiating activity, the defining characteristic of cancer stem cells. We therefore compared the ability of the ALDH^high^ cells and ALDH^low^ cells to form colonies on soft agar. The ALDH^high^ and ALDH^low^ subpopulations of TC71 and MHH-ES cells were collected, plated on soft agar, and allowed to grow for two weeks. The ALDH ^high^ cells gave rise to significantly more colonies than did the ALDH^low^ cells (p = 0.012; Figure 5A). The colonies formed by the ALDH^high^ cells were also substantially larger than the few colonies formed from ALDH^low^ cells – 49.3% of the colonies formed by ALDH^high^ cells were larger than 150 µm and 30.1% were larger than 200 µm, while only 13.1% of the colonies formed by the ALDH^low^ cells were larger than 150 µm and none were larger than 200 µm.

The ability to form spherical aggregates (“sarcospheres”) when cultured under non-adherent conditions is also a characteristic of cancer stem cells. ALDH^high^ and ALDH^low^ cells were isolated from the TC71 cell line, resuspended in supplemented Mesencult media, and plated on ultra low attachment plates. After one week, spherical aggregates ≥16 cells were counted. As anticipated, ALDH^high^ cells gave rise to approximately 4–5-fold more spheres than the ALDH^low^ cells, a statistically significant difference (p = 0.0015; Figure 5B). Similar results were found using MHH-ES, SK-ES-1, and A4573 cells as well (data not shown).

When we assayed the clonogenic activity (in soft agar) and sphere forming ability (in Mesencult) of cells isolated from one of the primary ESFT xenografts described above, ALDH^high^ cells gave rise to significantly more colonies and spheres than the ALDH^low^ cells (p<0.005 for sphere formation; Figure 5C and data not shown); in fact, the ALDH^low^ subpopulation from the xenograft was completely devoid of sphere forming activity, although the initial viability of both cell populations, as assessed by Trypan blue exclusion, was similar. The sphere formation assay was also conducted with cells from another xenograft (Figure 5D), sorted to exclude contaminating murine cells with high levels of ALDH expression (see Figure 3). Exclusion of these contaminating cells did not alter the differential sphere forming activity (Figure 5). In this case, 5,000 cells were plated in triplicate. The ALDH^high^ cells generated 34.67±2.4 spheres per well, significantly more than the unsorted cells (11.67±0.88; p = 0.0008) or the ALDH^low^ cells (0.667±0.33; p = 0.0002).

**Figure 6:**
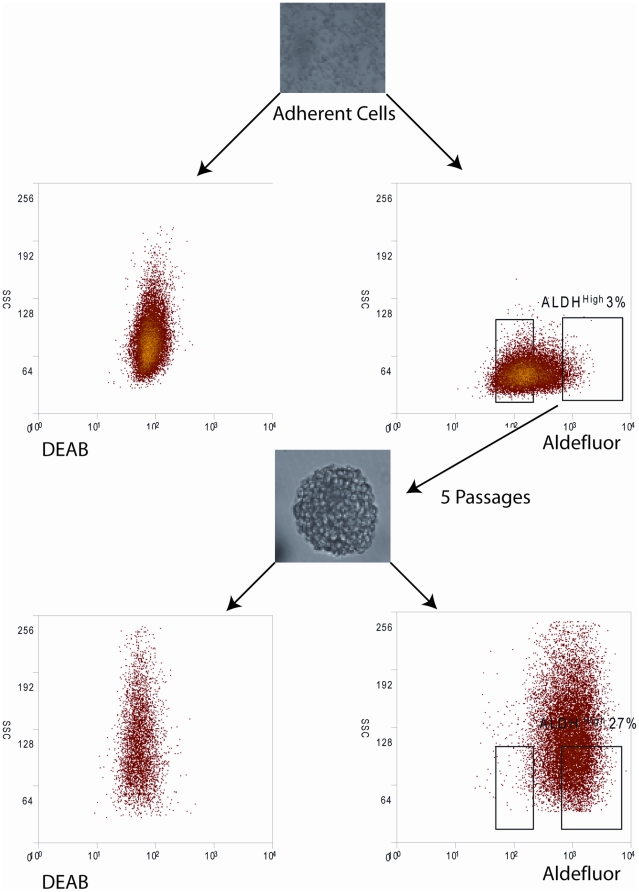
Passaging as sarcospheres enriches for ALDH^high^ cells. TC71 cells were grown under standard, adherent conditions. The cells with the highest 3% of ALDH activity were isolated and replated in Mesencult under nonadherent conditions. The resulting sarcospheres were disaggregated into single cells, sorted, and the ALDH^high^ cells replated under identical conditions. After 5 such passages, cells from disaggregated sarcospheres were analyzed by the Aldefluor assay. The initial gate, which encompassed only 3% of the starting cells, included 27% of the cells passaged as sarcospheres.

Finally, we investigated whether growing TC71 cells as sarcospheres would enrich for cells with high levels of ALDH expression. The ALDH^high^ population (in this case defined as the cells with the highest 3% of ALDH activity; Figure 6) from adherent TC71 cells was collected and plated under nonadherent conditions in Mesencult as described above. The resulting sarcospheres were collected, disaggregated, and replated in Mesencult under nonadherent conditions. After 5 such passages, cells were analyzed by the Aldefluor assay. In the primary adherent population, ALDH^high^ cells represented only 3% of the population, however after the 5^th^ passage, cells with an amount of ALDH activity, as determined by fluorescence intensity, equivalent to the starting cells constituted 27% of the total (Figure 6), a 9-fold increase. Similar results were seen with SK-ES-1 cells and A4573 cells (data not shown).

### ALDH^high^ but not ALDH^low^ cells are capable of reconstituting a heterogeneous population *in vitro*

**Figure 7:**
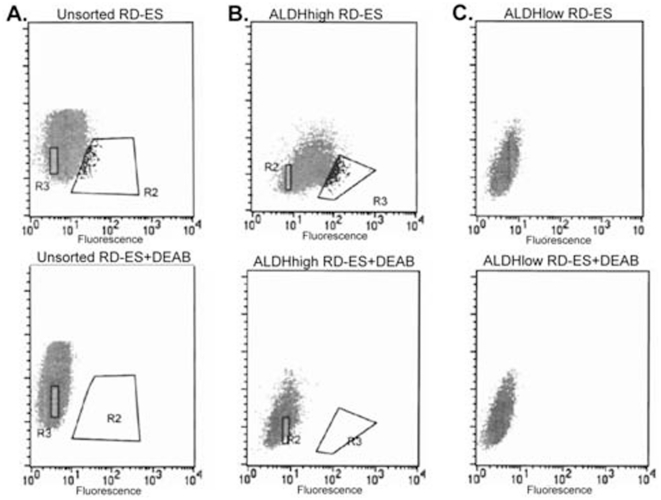
Self renewal ability of ALDH^high^ cells. (A) RD-ES cells were incubated with Aldefluor alone (top) or in the presence of DEAB (bottom). The ALDH^high^ (R2) and ALDH^low^ (R3) populations were isolated and cultured for 2 weeks. As described in the Methods section, these gates were chosen to isolate the cells with the highest and lowest 2% of ALDH activity. Cells were then incubated with Aldeflour and reanalyzed by FACS. (B) The ALDH^high^ cells give rise to both ALDH^high^ (gate R3) and ALDH^low^ (gate R2) populations. (C) The ALDH^low^ cells only regenerated an ALDH^low^ population. In both panels B and C, analysis was performed without (top) or with (bottom) DEAB, just as in panel A.

A defining property of stem cells is their ability to undergo asymmetric division, resulting in both a self renewing population of stem cells and a more differentiated non-stem cell population. To determine whether ALDH^high^ cells are capable of regenerating such a heterogeneous population, ALDH^high^ and ALDH^low^ cells were isolated from the RD-ES cell line and were cultured separately at similar density. After two weeks, we analyzed the cultured populations using the Aldefluor assay. The ALDH^high^ cells gave a similar profile to the parent population, with the majority of cells ALDH^low^ and only a small fraction being ALDH^high^. In contrast, ALDH^low^ cells were unable to generate ALDH^high^ cells (Figure 7). Similar results were obtained with the TC71 cell line (data not shown). The ability of the ALDH^high^ cells to re-generate a heterogeneous population of cells supports the hypothesis that high ALDH activity identifies a population enriched for ESFT stem cells.

### ALDH^high^ cells are enriched for expression of the “stem cell genes”

**Figure 8:**
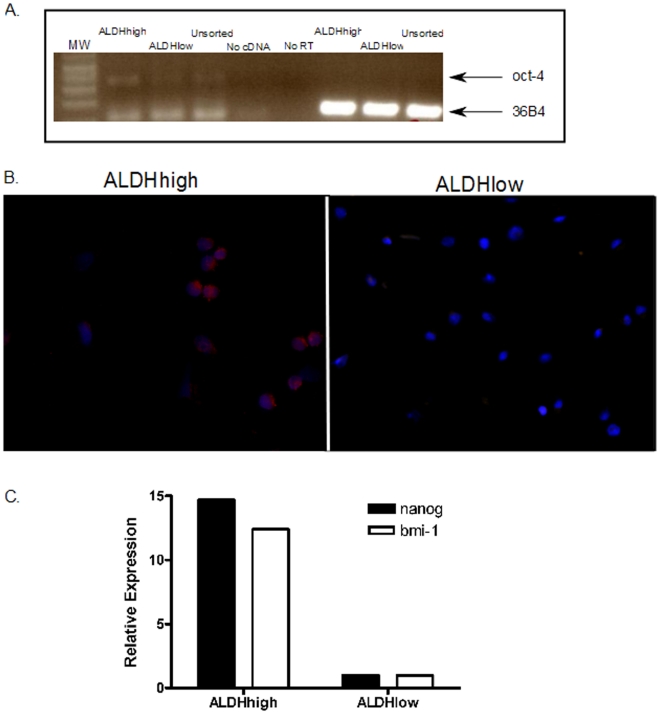
Expression of “stemness” genes in ALDH^high^ and ALDH^low^ cells. TC71 cells were treated with Aldefluor, and ALDH^high^, ALDH^low^, and unsorted cells were isolated. (A) Cells were analyzed for *oct-4* expression by RT-PCR using primers reported by Liedtke et al to not amplify known *oct-4* pseudogenes [29]. Expression of ribosomal RNA 36B4 is shown as a positive control. No cDNA and No RT negative controls are also shown. The lower band in the first 3 (*oct-4*) lanes probably represents primer dimers. (B) ALDH^high^ and ALDH^low^ TC71 cells were evaluated for Oct-4 protein expression by immunocytochemistry. The blue color represents DAPI staining (which stains all cell nuclei), and the red staining reflects Oct-4 expression. (C) RNA from ALDHhigh and ALDHlow TC71 cells was tested for *nanog* and *bmi-1* expression by quantitative RT-PCR. Each of these “stem cell genes” was expressed at significantly higher levels in ALDH^high^ cells than in ALDH^low^ cells.

We investigated whether ALDH^high^ cells are enriched for expression of genes that have been postulated to play key roles in stem cell biology, such as the polycomb group gene *bmi-1* [26], the POU DNA binding domain-containing gene *oct-4* [27], and *nanog*, a key regulator of stem cell pluripotency [28], relative to the ALDH^low^ population. We isolated the ALDH^high^ and ALDH^low^ subpopulations from TC71 cells and measured *oct-4* expression using primers specifically designed to exclude the numerous *oct-4* pseudogenes [29]. Qualitative PCR shows significant *oct-4* expression in the ALDH^high^ population, with less expression in the unsorted cells, and only minimal expression in the ALDH^low^ cells (Figure 8A). We confirmed this differential expression using immunocytochemistry. ALDH^high^ and ALDH^low^ TC71 cells were subjected to immunocytochemistry using an anti-Oct-4 antibody. Most of the ALDH^high^ cells were stained with the antibody, but no staining was seen in the ALDH^low^ cells (Figure 8B). We investigated *bmi-1 and nanog* RNA expression using quantitative RT-PCR, with *β2-microglobulin* expression as a control. We detected a 12.4-fold increase in *bmi-1* mRNA levels in ALDH^high^ compared to ALDH^low^ cells and a 14.7-fold increase in *nanog* mRNA(Figure 8C).

### ALDH^high^ cells exhibit tumor initiating activity *in vivo*

**Figure 9:**
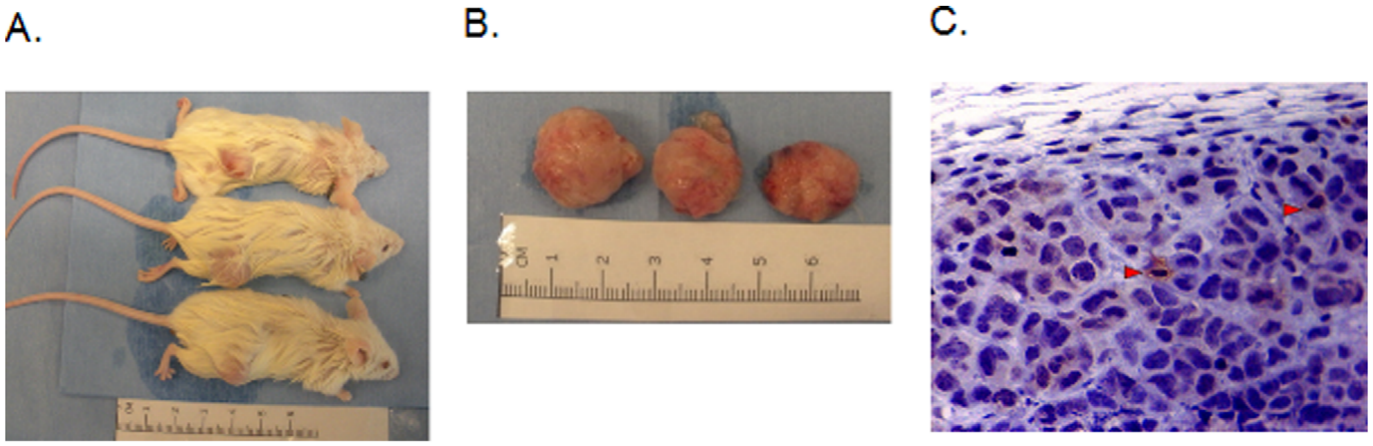
Analysis of tumors grown from ALDH^high^ ESFT cells. (A) Mice with tumors growing from ALDH^high^, but not ALDH^low^, MHH-ES cells injected subcutaneously in the flank. In each case, ALDH^high^ cells were injected in the right flank, and ALDH^low^ cells were injected in the left flank. (B) Gross appearance of resected tumors. (C) Immunohistochemical analysis of MHH-ES-derived tumors. Paraffin-embedded sections were evaluated for ALDH expression by immunohistochemistry, and counterstained with hematoxylin to evaluate tumor histology. Tumors are comprised of small round blue cells, as is typical of ESFT. The brown staining shows the rare ALDH-expressing tumor cells (indicated by red arrowheads), despite the fact that the cells injected into the mouse uniformly expressed high levels of ALDH.

In addition to self renewal, the other defining characteristic of a cancer stem cell is tumor initiation. To investigate the tumorigenicity of ALDH^high^ cells *in vivo*, we isolated ALDH^high^ and ALDH^low^ TC71 cells and injected them subcutaneously in the flanks of individual NOD/SCID/IL-2Rγ^null^ (NOG-SCID) mice. Unsorted cells were injected as a control. After 6–10 weeks, tumors were detected in the flank region with an average size of 10×12 mm. Tumor formation was seen after injection of as few as 160 ALDH^high^ cells (Table 1). In contrast, only 9 out of 13 of the mice injected with 800,000 ALDH^low^ cells and none of the mice injected with 80,000 or fewer ALDH^low^ cells developed a tumor, suggesting that this population was relatively depleted of tumor initiating cells. Injections of 800,000 unsorted cells reliably caused tumor formation, and 3 of 7 mice injected with 80,000 unsorted cells also developed a tumor. Thus, in TC71 cells, tumor initiating cells represent approximately 0.6% of the ALDH^high^ population and less than 10^−5^ of the ALDH^low^ population. Similar data were obtained using MHH-ES cells (Table 1). Importantly, tumors that arose from ALDH^high^ cells from both cell lines could be serially transplanted into secondary and tertiary recipients, consistent with the presence of ESSC in this population. Gross tumor appearance and histology were similar for tumors arising from the unsorted cells, the ALDH^low^ cells and the ALDH^high^ cells (Figure 9). Immunohistochemistry demonstrated that only a small minority (∼1%) of tumor cells expressed a high level of ALDH, even in tumors that arose from implantation of purified ALDH^high^ cells (Figure 9C), supporting the hypothesis that these represent true cancer stem cells, able to generate serially-transplantable tumors that contain mostly ALDH^low^ non-stem cells.

**Table 1:** Tumor Initiating Capacity of Various Cell Populations.

	TC71 Cells					MHH-ES Cells		
	Cell Number					Cell Number		
	800,000	80,000	8,000	800	160	8,000	800	160
	(Tumors/Mouse)					(Tumors/Mouse)		
Unsorted	3/3	3/7	0/5			0/5		
ALDH^high^	4/4	4/4	8/15	6/10	6/6	5/5	5/5	4/5
ALDH^low^	9/13	0/4	0/4			0/5	0/5	0/5
CD133+				2/2	0/2		0/3	0/5

### ALDH provides superior enrichment for stem cells compared with CD133

The cell surface antigen CD133 has been proposed to be a stem cell marker, and CD133-positive ESFT cells have tumor initiating activity [30]. We therefore compared clonogenic and tumor initiating activities of our ALDH^high^ cells with those of CD133-positive cells. We isolated ALDH^high^ and CD133-positive TC71 cells by FACS (Figure 5E) and cultured 1000 cells/well of each population on ultra low attachment plates supplemented with Mesencult to compare their sphere forming ability. After one week in culture, although the CD133-positive cells did give rise to more spheres than the CD133-negative/ALDH^low^ cells (p = 0.001; Figure 5F), the ALDH^high^ cells gave rise to significantly more sarcospheres than the CD133-positive cells (p = 0.015). In addition to clonogenic activity, we also compared tumor initiating activity of CD133-positive and ALDH^high^ cells. Although as few as 800 CD133-positive cells were sufficient to give rise to tumors in NOG-SCID mice, unlike the ALDH^high^ cells, injection of 160 CD133-positive cells did not result in tumor formation (Table 1). Similar results were seen using MHH-ES cells (Table 1). Thus, both *in vitro* and *in vivo* assays demonstrate that, in both TC71 and MHH-ES cells, the ALDH^high^ population of cells is more enriched for stem cell activity than the CD133-positive cells.

### ALDH^high^ cells are resistant to chemotherapy

**Figure 10:**
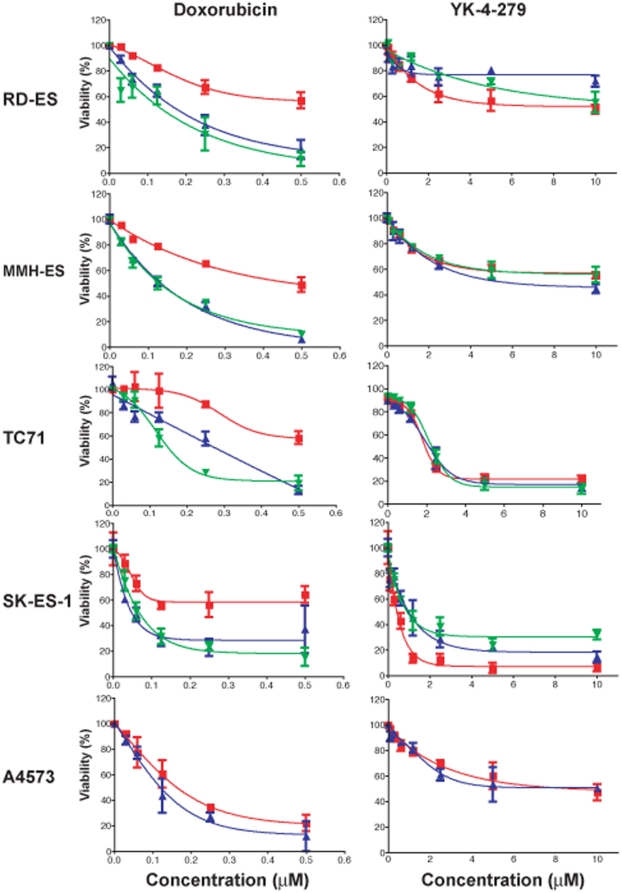
Relative chemoresistance of ALDH^high^ cells. Sorted ALDH^high^ and ALDH^low^ TC71, A4573, SK-ES-1, MHH-ES, and RD-ES cells, as well as unsorted cells, were treated for 48 hours with increasing concentrations of either doxorubicin (left) or YK-4-279 (right). Surviving cells were quantified based on their staining with DAPI but not PI. Experiments were performed in triplicate, and multiple independent FACS separations were analyzed. In each panel, the red line is the survival curve for ALDH^high^ cells, the blue line is shows the ALDH^low^ cells, and the unsorted cells are represented by the green line. Bars = SEM.

The cancer stem cell hypothesis proposes that the discrepancy between treatment response and patient survival noted in most cancer types reflects an inherent resistance of the cancer stem cells to chemotherapy. We therefore investigated whether ALDH^high^ ESFT cells are resistant to chemotherapy, as would be expected if this population is enriched for cancer stem cells. ALDH^high^ and ALDH^low^ cells were isolated from TC71, A4357, MHH-ES, SK-ES-1, and RD-ES cell lines and incubated with increasing doses of doxorubicin, one of the most important chemotherapy drugs for the treatment of ESFT. Cell survival was evaluated by counting viable cells remaining after 48 hours. In all 5 cell lines, ALDH^low^ cells showed the same dose dependent decrease in cell viability seen with the unsorted cells, whereas in 4 of the 5 lines, the ALDH^high^ cells were significantly more resistant to doxorubicin over the dose range tested (Figure 10 left panels). Similar results were obtained using etoposide in place of doxorubicin (data not shown).

**Figure 11:**
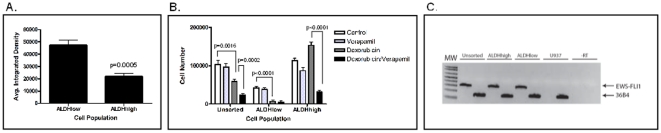
Efflux pumps may mediate relative chemoresistance of ALDH^high^ cells. (A) ALDH^high^ and ALDH^low^ TC71 cells were incubated with Hoechst 33342 for 45 minutes, followed by a 45 minute wash out period. Average cellular fluorescence was quantified as relative fluorescence units (RFU). Assay was performed in triplicate at least three times. Bars = SEM. (B) Unsorted TC71 cells, as well as ALDH^high^ and ALDH^low^ cells, were incubated for 48 hours with 500 nM doxorubicin, 10 µM verapamil, or both. Surviving cells were quantified based on Trypan blue exclusion. Assays were performed in triplicate at least three times. Bars = SEM. (C) Expression of EWS-FLI1 in ALDH^high^ cells as detected by RT-PCR. RNA was isolated from ALDH^high^, ALDH^low^, and unsorted TC71 cells. RT-PCR was performed using primers specific for EWS-FLI1 and for ribosomal RNA 36B4 as a positive control. Human myelomonocytic leukemia cell line U937 serves as a negative control.

One of the proposed mechanisms by which cancer stem cells resist chemotherapy is by enhanced expression of ATP-binding cassette (ABC) transport proteins, which are responsible for drug efflux. Higher expression of ABC transport proteins in stem cells compared to non-stem cells results in relative resistance of the stem cells to the toxic effects of chemotherapy drugs compared with the bulk population. We address this aspect of stem cells using a functional evaluation of fluorescent dye efflux. ALDH^high^ and ALDH^low^ cells isolated from the TC71 cell line were incubated with Hoechst 33342 for 45 minutes, followed by a 45 minute wash-out. After the wash-out, cells were photographed under UV light and the relative fluorescence units (RFU) quantified. This assay revealed a significant increase in dye efflux by the ALDH^high^ cells compared with the ALDH^low^ cells. The ALDH^high^ population had an RFU of 22045+/−2583 compared with 47386+/−3756 in the ALDH^low^ cells (Figure 11A). This difference is highly statistically significant (p = 0.0005).

The calcium channel blocker, verapamil, blocks the activity of ABC transporter proteins, and is used to corroborate ABC transporter function [31]. If differential expression of these drug pumps mediates the relative resistance of ALDH^high^ cells to cytotoxic chemotherapy, then this resistance should be reversed by concurrent treatment with verapamil. To test this hypothesis, ALDH^high^ and ALDH^low^ cells isolated from the TC71 cell line were incubated for 48 hours with 500 nM doxorubicin with or without 10 µM verapamil. The doxorubicin killed 55% of the unsorted cells (p = 0.0016 compared with untreated controls), and the addition of verapamil significantly augmented this toxicity – killing 40% more cells (p = 0.0002). The ALDH^low^ cells were even more sensitive to doxorubicin, with 500 nM killing 85% of the cells in 48 hours (p<0.0001 for the difference between control and doxorubicin-treated), and the addition of verapamil did not cause a significant increase in the efficacy of doxorubicin against this population. As previously observed, although doxorubicin alone did not affect the survival of the ALDH^high^ cells, there was a statistically significant 80% decrease in the viability of ALDH^high^ cells treated with both 10 µM verapamil and 500 nM doxorubicin (p<0.0001 between doxorubicin alone and doxorubicin plus verapamil; Figure 11B).

### Standard cytotoxic chemotherapy drugs do not inhibit clonogenic activity of ALDH^high^ cells

**Figure 12:**
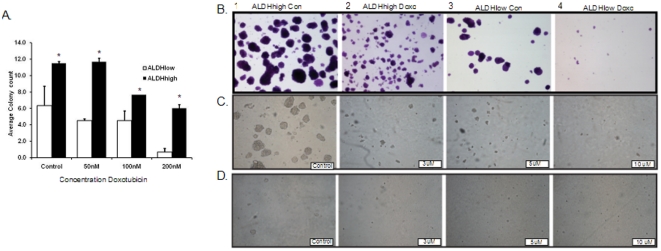
ALDH^high^ clonogenic activity is relatively resistant to doxorubicin but remains sensitive to EWS-FLI-1 inhibition. A) ALDH^high^ and ALDH^low^ TC71 cells were plated at 50,000 cells per well on soft agar in the presence or absence of the indicated concentration of doxorubicin. After 14 days, colonies were counted in 3 high power fields per well. Data represents average colony count from a representative experiment done in duplicate wells. Bars = SEM. The asterisk (*) indicates conditions where the difference between ALDH^high^ and ALDH^low^ cells reached statistical significance with a p value <0.05. Representative images from ALDH^high^ (B 1 and 2) and ALDH^low^ (B3 and 4) colonies grown on soft agar stained with crystal violet. Cells were treated with nothing (B1 and B3) or 200 nM doxorubicin (B2 and B4). Magnification: 100×. C and D) Representative images from soft agar colony formation assay done in the presence of different doses of YK-4-279 are shown. ALDH^high^ (C) and ALDH^low^ (D) TC71 cells were plated at 50,000 cells/well and treated with nothing or the indicated doses of the compound for 7 days. Magnification: 100×.

Our *in vivo* tumor initiation data demonstrate that only a fraction of the ALDH^high^ cells have tumor initiating (stem cell) activity. The data presented above demonstrate that ALDH^high^ cells are relatively resistant to chemotherapy, but they do not prove that the resistant cells are the ones with the stem cell phenotype. The cancer stem cell hypothesis predicts that the chemotherapy-resistant ALDH^high^ cells should retain stem cell activity. To test this, we assessed the clonogenic activity of ALDH^high^ and ALDH^low^ cells treated with doxorubicin. ALDH^high^ and ALDH^low^ TC71 cells were seeded separately on soft agar and treated with varying concentrations of doxorubicin. After two weeks, colonies were stained and counted in individual wells. At each doxorubicin dose we detected both larger and more numerous colonies arising from ALDH^high^ compared with ALDH^low^ cells (Figure 12A). Moreover, although ALDH^low^ cells showed a dose dependent decrease in clonogenicity and inhibition of almost all colony growth by 200 nM doxorubicin, the clonogenic activity of ALDH^high^ cells was unaffected by low doses of the drug and decreased only modestly in response to 200 nM doxorubicin (Figure 12B). These data suggest that the ALDH^high^ cells which resist the cytotoxic effects of doxorubicin retain clonogenic activity.

### ALDH^high^ cells retain sensitivity to EWS-FLI1 inhibition

We next tested our hypothesis that inhibition of EWS-FLI1 activity is toxic to ESFT CSC that are resistant to standard cytotoxic chemotherapy. One critical function of EWS-FLI1 involves a physical interaction with RNA helicase A (RHA). A small molecule, YK-4-279, has been developed to specifically disrupt this interaction [19]. This compound induces apoptosis in ESFT cells both *in vivo* and *in vitro*. We investigated whether YK-4-279 is toxic to the ALDH^high^ subpopulation of ESFT cells. In order for YK-4-279 to have an effect on ALDH^high^ cells, these cells must express EWS-FLI1. Using RT-PCR we confirmed EWS-FLI1 expression in ALDH^high^ cells (Figure 11C). To assess the sensitivity of ESFT CSC to YK-4-279, ALDH^high^ and ALDH^low^ cells were isolated from RD-ES, MHH-ES, TC71, SK-ES-1, and A4573. Isolated cell populations were incubated with increasing doses of either YK-4-279 or doxorubicin. Cell survival was evaluated by counting viable cells remaining after 48 hours using the Trypan blue exclusion assay. The ALDH^high^ and ALDH^low^ cells showed identical dose dependent decreases in cell viability when treated with YK-4-279 (Figure 10, right panels). In contrast, ALDH^high^ cells obtained from TC71, SK-ES, RD-ES, and MHH-ES were relatively resistant to doxorubicin when compared to ALDH^low^ cells (Figure 10, left panels).

To fully assess whether YK-4-279 eradicates ESFT stem cell activity, ALDH^high^ and ALDH^low^ TC71cells were plated in soft agar in the presence or absence of YK-4-279. At doses as low as 3 µM, the clonogenic activity of the ALDH^high^ cells was inhibited. One week following treatment we detected a dose dependent decrease in both the number and size of colonies from ALDH^high^ and ALDH^low^ cells, and by two weeks almost no viable colonies were detected, only clumps of cell debris (Figure 12C and D). Thus, ALDH^high^ cells retain sensitivity to YK-4-279 induced EWS-FLI1 inhibition, as reflected by both direct cytotoxicity and by loss of clonogenic activity. Notably, treatment of ALDH^high^ cells with doxorubicin does not eliminate clonogenic activity, but treating these cells with YK-4-279 does (Figure 12).

## Discussion

ESFT is a chemotherapy-sensitive cancer, and even patients who present with metastatic disease commonly achieve remission. Despite this, 25% of patients who present with a localized tumor and 70% of patients who present with metastases will develop a recurrence and ultimately die of their disease [1], [2], [3]. The presence of chemotherapy-resistant CSC has been put forth as one explanation for this discrepancy between response to treatment and patient survival. Not every tumor type has been shown to follow the CSC model [8], so we and others have sought evidence for the existence of a Ewing's sarcoma stem cell. Our work has demonstrated that the subpopulation of ESFT cells that express the highest levels of ALDH have several characteristics of stem cells, including the capacity to generate a heterogeneous population, *in vitro* clonogenic activity and *in vivo* tumorigenic activity. As predicted, these cells are resistant to standard cytotoxic chemotherapy; however, we have demonstrated that these cells retain sensitivity to a small molecule inhibitor of EWS-FLI1.

Several approaches have been taken to identify CSC from other tumor types, including evaluation of immunophenotype and various functional assays. We chose to pursue ALDH expression because high expression of ALDH is thought to provide a survival advantage in a toxic cellular microenvironment, and studies have demonstrated high ALDH expression in CSC from breast and colorectal carcinomas. No single assay is likely to yield a pure population of CSC, but our data demonstrate that as few as 160 ALDH^high^ cells can form a tumor in immune deficient mice suggests that ALDH expression allows substantial enrichment of this population.

Suva et al recently showed that CD133 expression marks a population of Ewing's sarcoma cells with tumor initiating activity [30]. Our experiments extend that initial observation of a ‘stem-like’ population in Ewing's sarcoma in several important ways and have identified a more consistent tumor-initiating cell population. Although both isolation methods (CD133 expression and Aldefluor) result in a population enriched for cells able to reconstitute tumors when implanted in immunodeficient mice, our work demonstrates that the unique ALDH^high^, low side scatter population of cells is more enriched for cells with clonogenic activity in vitro and tumor initiating activity in vivo than the CD133-positive cells. In addition, the ALDH^high^ population of cells expresses high levels of several genes associated with a stem cell phenotype (*bmi-1*, *oct-4*, and *nanog*) and can reconstitute the heterogeneous parental population *in vitro* and *in vivo*. Our most clinically important observation, distinct from prior publications, is that the ALDH^high^ population is relatively resistant to cytotoxic chemotherapeutic agents. Interestingly, Jiang et al. recently reported that there is almost no correlation between CD133 expression and resistance to chemotherapy in ESFT cell lines ([32]). We demonstrated that, unlike CD133+ cells, in 4 of the 5 cell lines tested, ALDH^high^ cells are resistant to both doxorubicin and etoposide. Intriguingly, the one cell line that did not show relative resistance of ALDH^high^ cells to doxorubicin, A4573, has previously been demonstrated to be more relatively more resistant to this agent than other ESFT cell lines [33]. Most important in our findings is that, in contrast to the differential resistance of ALDH^high^ cells to standard cytotoxic chemotherapeutic agents compared with ALDH^low^ cells, ALDH^high^ cells retain sensitivity to the EWS-FLI1 targeted inhibitor, YK-4-279. This not only identifies a potential clinical strategy to prevent tumor recurrence, but also suggests that ESFT repopulating cells are still dependent on the activity of the pathognomonic tumor-associated transcription factor EWS-FLI1. In addition to many previous publications that validate EWS-FLI1 as a therapeutic target, we now show that targeting EWS-FLI1 will potentially access ESFT cells that become resistant to conventional chemotherapy.

One limitation to our work is our inability to use primary tumor samples as a source of ALDH^high^ cells to confirm that such cells isolated directly from patients have a stem cell phenotype. ESFTs are most often diagnosed by needle biopsy and not resected until after administration of neoadjuvant chemotherapy, such that adequate numbers of fresh cells could not be obtained in primary patient samples. Thus it is not possible to obtain sufficient material from patients to verify our cell line and xenograft findings. We have attempted to address this limitation by evaluating patient biopsy specimens for ALDH expression using immunohistochemistry (Figure 4). Results of this analysis are consistent with ALDH expression marking a stem cell population. Future studies will be designed to evaluate the impact of the percentage of ALDH positive cells on clinicopathologic features of the disease.

The clinical significance of CSC is their inherent resistance to chemotherapy as CSCs from other solid tumors are chemoresistant. Following chemotherapy, Dylla et al., detected enrichment of CSC in colorectal carcinoma xenografts [34]. In squamous cell cancers, side population cells (cells that exclude the fluorescent dye Hoechst 33342) maintain clonogenicity in the presence of cytotoxic drugs [35]. In another study, side population cells isolated from primary neuroblastoma exhibited an enhanced capacity to expel cytotoxic drugs [36]. Multiple mechanisms have been proposed to explain the chemoresistance of CSC. The best characterized of these is high expression of ABC transporter proteins which mediate drug efflux [37]. ALDH itself also contributes to chemoresistance – it functions as a detoxification enzyme for chemotherapeutic drugs such as cyclophosphamide [38]. Our data demonstrate that ESSC are resistant to two of the standard agents used to treat ESFT – doxorubicin and etoposide. Our dye efflux assay suggests that ESSC have relatively higher transport protein activity than the bulk population, and chemoresistance is reversed by verapamil, an inhibitor of ABC transport proteins. Thus, drug efflux pump activity may be an important mechanism of ESSC chemoresistance. Future investigations will evaluate drug efflux pump protein expression in ESSC populations.

The relatively static survival rates for patients with Ewing's sarcoma, especially those with metastatic disease, despite intensification of standard chemotherapy [1] suggests that novel, biologically based treatment approaches must be developed. Our small molecule, YK-4-279, represents a different approach to targeted therapy for ESFT. This compound disrupts a critical interaction between EWS-FLI1, the protein product encoded by the fusion gene resulting from the t(11;22) translocation, and a heterologous binding protein, RHA. Disruption of this interaction induces apoptosis in ESFT cells [19]. Our data show that although ESSC are resistant to standard cytotoxic agents, they retain sensitivity to YK-4-279. Combined with evidence that YK-4-279 inhibits the growth of ESFT xenografts in mice, these data provide support for the idea that small molecules that disrupt critical protein-protein interactions involving EWS-FLI1 represent a promising therapeutic approach for patients with high risk disease for whom standard chemotherapy is inadequate.

## Materials and Methods

### Cell lines and xenograft model

ESFT cell lines TC-71, MHH-ES, SK-ES-1, A4573, and RD-ES were obtained from ATCC (Manassas, VA). Cells were cultured at 50–70% confluence in RPMI-1640 medium supplemented with 10% fetal bovine serum (Invitogen, Grand Island, NY) and 1% penicillin/streptomycin (Quality Biological Inc., Gaithersburg, MD). Transplantable xenografts of primary human ESFT were a kind gift from Dr. Chand Khanna and Dr. Lee Helman (National Institutes of Health). The creation of these xenografts was approved by the Institutional Review Board of the National Institutes of Health, and patients gave informed consent for their creation. Xenografts were re-engrafted into six to eight week old NOG-SCID mice (Jackson Laboratory, Bar Harbor, Maine; colony maintained at JHMI). Mice were anesthetized with a single intraperitoneal dose of 240 mg/kg Avertin (Sigma, St. Louis, MO), two 3 mm tumor fragments were implanted adjacent to the right proximal tibia bone after creating a small incision in overlying muscle, liquid tissue adhesive (NEXABAND®, Abbott Laboratories, North Chicago, IL) was applied to seal the muscle, and the skin wound was closed with 9 mm surgical clips. Mice were maintained in a pathogen-free environment and monitored weekly for tumor growth. After 10–12 weeks, mice were sacrificed; tumors were harvested and prepared into single cell suspension for further experiments. All procedures were performed according to a protocol approved by the Johns Hopkins Animal Care and Use Committee.

To create single cell suspension, tissue samples were mechanically minced with a scalpel and further dissociated in 0.25% trypsin (Wheaton Sciences, USA). Cells were allowed to recover overnight at 37°C and the next day were analyzed by FACS. To eliminate cells of mouse origin, a biotin labeled mouse anti-mouse H-2K[d] antibody (SF1-1.1, BD Pharmingen) was used at 1∶200 dilution in Aldefluor assay buffer containing 2% FBS for 20 minutes on ice(Reference- Ginestier C, Dontu G, 2007). The cells were subsequently labeled with APC-conjugated Streptavidin (SAV-APC, eBioscience) at 0.06 µg per sample for 20 minutes on ice and analyzed using the BD- FACSAria flow cytometer.

### Aldeflour assay and Fluorescence-activated cell sorting (FACS)

The Aldefluor® assay (Stem Cell Technologies, Vancouver, BC) was performed according to the manufacturer's instructions. ALDH^high^ cells were identified in ESFT cell lines and xenografts by comparing the same sample with and without the ALDH inhibitor DEAB. Cells were analyzed and sorted using FACSAria and FACSDiva software (BD Biosciences, Franklin Lakes, NJ). Dead cells were excluded based on light scatter characteristics and gates were set to select the brightest and dimmest 2–3% of the analyzed population on average. Viability of sorted cells was 55–65% as tested by Trypan blue dye exclusion (Invitrogen) or by exclusion of PI. In some experiments, cells were labeled with PE-conjugated anti-human CD133/2 (Miltenyi Biotec, Auburn, CA.) after being incubated with Aldefluor. Samples were incubated in the dark with 5 µg/ml of the antibody or the isotype control IgG for 10 min at 4°C, then washed, resuspended, and analyzed by FACS.

### Immunohistochemistry

Paraffin-embedded ESFT tissue samples were deparaffinized in xylene and rehydrated in graded alcohol and rinsed in 1× PBS. Antigens were retrieved by boiling samples for 12 minutes in citrate buffer, pH 6 (Invitrogen). Nonspecific binding sites were blocked using 1 mL PBS containing 5% goat serum and 1% bovine serum albumin (BSA). The sections were incubated overnight at 4°C in a humidor with monoclonal antibody to ALDH1 (1∶100; BD bioscience, clone 44), diluted with 1% goat serum, 0.2% BSA and 0.3% Triton X-100 in PBS (pH 7.4), followed by washing with PBS. Sections were then incubated with peroxidase-conjugated secondary antibody (Jackson Immunoresearch Laboratories, USA) of appropriate specificity. 3,3′-diaminobenzidine (DAB, Pierce) was used as substrate for peroxidase and counterstaining was performed with modified Harris hematoxylin solution (Sigma). Sections were dehydrated by passage through graded alcohol concentrations and finally xylene. Cover slips were mounted using DPX (Sigma). Completed immunostaining was visualized using microscopy (Nikon E600), and photographed with digital camera (Nikon DXM1200F; ACT-1 software).

For immunocytochemical detection of Oct4, TC71 cells were pelleted on slides using a cytospin. Cells were fixed with 4% paraformaldehyde for 20 minutes at room temperature, washed with PBS, and blocked in PBS with normal goat serum for 1 hour at room temperature. After blocking, cells were stained overnight with primary antibody against Oct 4 (Clone C10, Santa Cruz Biotechnology, Inc.) at a 1∶100 dilution in PBS with 5% normal goat serum and 0.02% Triton X-100 at 4°C. The next day, cells were washed with PBS and stained with Alexa Fluor 555 goat anti-mouse antibody (Invitrogen) at 1∶400 dilution. After secondary labeling, cells were washed in PBS and mounted in Prolong Gold with DAPI.

### Analysis of ALDH immunohistochemical staining

Quantification of ALDH expression was performed using FRIDA (FRamework for Image Dataset Analysis; [39], [40]), a custom open source image analysis software package (available at http://sourceforge.net/projects/fridajhu/) for the analysis of RGB color images generated from scanning of tissue microarray slides. Hue Saturation and Brightness (HSB) segmentation ranges for DAB brown staining and hematoxylin alone (cells not staining brown) were defined by creating different color masks. Numbers of cells were counted by using particle count filter set with size limitation. The percentage of ALDH+ cells were calculated by using No. of DAB labeled cells divided by the sum of the DAB labeled and the hematoxylin labeled cells ×100.

### Soft agar colony formation

To test clonogenic activity, sorted ALDH^high^ or ALDH^low^ cells from the TC71 cell line or from ESFT xenografts were seeded at a density of 10^3^ to 10^4^ cells per well in 6 well plates (Corning Inc, NY). Cells were cultured in 0.3% top agar (Invitrogen) prepared from a 1∶1 dilution of 0.6% bottom agar in RPMI-1640 medium supplemented with 10% FBS. After 1 hour, top agar was overlayed with 1 ml fresh media. Cells were maintained in a 37°C tissue culture incubator and after 2 weeks (6 weeks for cells from xenografts) all colonies ≥20 cells were counted in duplicate or triplicate wells using an inverted light microscope. To test the drug sensitivity of colony formation, ALDH^high^ or ALDH^low^ cells were plated at 50,000 cells/well and 24 hours later duplicate wells were treated with doxorubicin (Sigma, St. Louis, MO) or YK-4-279. After 2 weeks, colonies were stained with p-Nitro Blue Tetrazolium Chloride (USB, Cleveland, OH) and scored by light microscopy in 3 high powered fields/well. Colony size was quantified using MCID Elite image analysis software (MCID, Cambridge, UK).

### Sphere formation

Sorted ALDH^high^ or ALDH^low^ cells obtained from the TC71 and MHH-ES cell lines or from the ESFT xenografts were cultured in MesenCult (MSC Basal Medium) supplemented with human MSC Stimulatory Supplements (Stem Cell Technologies, Vancouver, BC). The indicated number of cells was seeded on ultra low attachment plates (Corning, Inc, NY) in triplicate wells. Spherical aggregates ≥16 cells were counted in individual wells after 1 week (2 weeks for cells from xenografts).

### *In vivo* tumorgenicity

Groups of 4–10 mice (six to eight week old NOG-SCID) were injected with either sorted or unsorted cells subcutaneously into the right flanks in a 1∶1 mixture of Matrigel and HBSS in a final volume of 200 µl. Mice were sacrificed when tumors reached 15 mm.

### Dye efflux assay

Sorted ALDH^high^ and ALDH^low^ TC71 cells were resuspended at 10^6^ cells/ml in RPMI-1640 medium containing 1% FBS and were incubated with 5 µg/ml Hoechst 33342 dye (B2261, bisBenzimide HOE 33342, Sigma-Aldrich) at 37°C for 45 minutes. Unincorporated dye was washed away using cold HBSS containing 2% BSA at 4°C. Subsequently, the cells were incubated in warm RPMI containing 1% FBS at 37°C for 45 minutes for dye efflux. Cells were centrifuged at 4°C and mounted on glass slides in PBS for microscopy using Nikon Eclipse Ti microscope. Photomicrographs were acquired immediately and analyzed using Image J software (National Institutes of Health, Bethesda, MD).

### Chemosensitivity assays

Cell lines were labeled with Aldeflour reagent. ALDH^high^ and ALDH^low^ cells were plated at similar density in RPMI-1640 medium with 10% FBS (2×10^3^ to 10^4^ cells per well in 6 well plates).After 24 hours, duplicate wells were treated with doxorubicin or etoposide (Sigma, St. Louis, MO). In some experiments, cells were also treated with 10 µM verapamil as an ABC transporter protein inhibitor. After 48 hours, cells in individual wells were collected, centrifuged and resuspended in 50 µl PBS. To assess viability, cells were stained with 4′,6-diamidino-2-phenylindole (DAPI) and propidium iodide (PI). Cells staining with DAPI and not PI were deemed viable and were counted.

### Reverse Transcription-Polymerase Chain Reaction (RT-PCR) and qPCR

RNA was extracted from ALDH^high^, ALDH^low^ or unsorted TC71 cells using the RNeasy Mini Kit according to manufacturer's instructions (QIAGEN Inc, Valencia, CA) reverse transcribed as previously described (Iscript Reverse Transcriptase Bio-Rad, Hercules, CA). For the PCR reaction we used Platinum Blue PCR SuperMix (Invitogen, Grand Island, NY), 1 µl cDNA and 31 cycles of 94°C for 15 s, 60°C for 30 s, 72°C for 30 s. The PCR product was separated by 1% agarose gel electrophoresis and visualized with ethidium bromide. For quantitative PCR, 1 µl cDNA was mixed with 12.5 µl SYBER Green SuperMix and appropriate primers. Quantitative PCR was performed using a standard two step amplification/melt protocol.

The EWS/FLI-1 primers were forward: TCCTACAGCCAAGCTCCAAGTC; reverse: ACTCCCCGTTGGTCCCCTCC. The *oct-4* primers were as published by Liedtke et al [29]. Primers for *bmi-1, nanog*, and β2microglobulin were obtained from SABiosciences (Frederick, MD).

### Statistical Analysis

Differences in clonogenic activity and sphere formation between ALDH^high^ and ALDH^low^ cells were tested for significance using a two-sided t test. Differences in chemosensitivity were analyzed by one-way ANOVA. All statistical analyses were performed with Prism 4 software (GraphPad Software, Inc.).
